# A *Puccinia striiformis* f. sp. *tritici* secreted protein activates plant immunity at the cell surface

**DOI:** 10.1038/s41598-017-01100-z

**Published:** 2017-04-25

**Authors:** Bayantes Dagvadorj, Ahmet Caglar Ozketen, Ayse Andac, Cian Duggan, Tolga Osman Bozkurt, Mahinur S. Akkaya

**Affiliations:** 1 Middle East Technical University, Biotechnology Program, Department of Chemistry, Dumlupinar Blvd., Cankaya, Ankara TR-06800 Turkey; 2 Imperial College London, Department of Life Sciences, London, SW7 2AZ UK

## Abstract

Pathogens secrete effector proteins to suppress host immunity, mediate nutrient uptake and subsequently enable parasitism. However, on non-adapted hosts, effectors can be detected as non-self by host immune receptors and activate non-host immunity. Nevertheless, the molecular mechanisms of effector triggered non-host resistance remain unknown. Here, we report that a small cysteine-rich protein PstSCR1 from the wheat rust pathogen *Puccinia striiformis* f. sp. *tritici* (*Pst*) activates immunity in the non-host solanaceous model plant *Nicotiana benthamiana*. PstSCR1 homologs were found to be conserved in *Pst*, and in its closest relatives, *Puccinia graminis* f. sp. *tritici* and *Puccinia triticina*. When PstSCR1 was expressed in *N*. *benthamiana* with its signal peptide, it provoked the plant immune system, whereas no stimulation was observed when it was expressed without its signal peptide. PstSCR1 expression in *N*. *benthamiana* significantly reduced infection capacity of the oomycete pathogens. Moreover, apoplast-targeted PstSCR1 triggered plant cell death in a dose dependent manner. However, in Brassinosteroid insensitive 1-Associated Kinase 1 (*SERK3*/*BAK1*) silenced *N*. *benthamiana*, cell death was remarkably decreased. Finally, purified PstSCR1 protein activated defence related gene expression in *N*. *benthamiana*. Our results show that a *Pst*-secreted protein, PstSCR1 can activate surface mediated immunity in non-adapted hosts and contribute to non-host resistance.

## Introduction

Surface localized pattern recognition receptors (PRRs) mediate pathogen associated molecular pattern (PAMP)-triggered immunity (PTI) against a variety of plant pathogens^1–3^. PTI initiates the generation of reactive oxygen species (ROS), synthesis of salicylic acid, plant defence gene expression, stomatal closure and callose accumulation^4^. In some cases, surface immune receptors stimulate localized programmed cell-death, known as the hypersensitive response (HR)^5^, although what determines the decision for cellular suicide remains unclear. Nevertheless, adapted pathogens can either evade or suppress PTI by secreting a wide range of effector proteins into the apoplast, cytoplasm, and other host subcellular compartments^6–8^. Apoplastic effectors can interact with surface proteins and other extracellular molecules^8^ such as defence-related enzymes to perturb their functions, enabling parasitism^9–13^. Some known apoplastic effectors include cell wall-degrading enzymes, toxins, ethylene inducing peptides, and small cysteine-rich (SCR) proteins. Since SCR effectors can form disulfide bonds, they are thought to be more stable in the harsh conditions of apoplast^8, 14^. Some effectors are known to stimulate cell death through surface localized immune receptors^8, 15–18^. Despite major progress made in previous decades, the biochemical functions and the host interactors of apoplastic effectors are largely unknown. Particularly in pathogenic fungi, it has been difficult to determine whether an effector is apoplastic, or host-translocated, due to the absence of canonical amino acid sequence motifs as seen in oomycete RXLR effectors^19, 20^. Discovering how effectors operate in different subcellular compartments is critical for understanding the mechanisms of host-pathogen interactions that will eventually lead to new ways of engineering plant disease resistance.

Wheat yellow rust disease, caused by *Puccinia striiformis* f. sp. *tritici* (*Pst*), is one of the major threats to global wheat production^21^. Although genomics studies helped identification of numerous candidate *Pst* effectors^22–24^, their modes of action are yet to be discovered. Dissecting the functions of effectors has been difficult due to absence of effective tools for functional gene analysis in crops^25, 26^. As an alternative, *N*. *benthamiana* serves as a model plant to study molecular plant-microbe interactions^27^. Recently, Petre *et al*. have reported that *N*. *benthamiana* is a feasible experimental tool to functionally analyse candidate effectors from *Pst*, a fungal pathogen of wheat^28^.

In this work, using *N*. *benthamiana* as a model system, we studied subcellular localization, function and response to pathogen infections of PstSCR1, which was previously predicted as a candidate effector^23^. Our data allowed us to conclude that PstSCR1 is an apoplastic effector of *Pst*, which is recognized in PAMP-triggered immunity. In non-adapted hosts, effectors can assist to explore the components of non-host resistance and discover novel participants of plant immunity.

## Results

### PstSCR1 is a *Puccinia* specific effector induced during infection

Fifteen candidate *Pst* effector gene expressed sequence tags (ESTs) were reported previously^23^. Among these, six have been further examined for developmental stage-specific gene expression^23^. The EST “GH737102” sequence that encodes PstSCR1 appeared as a full-length cDNA possessing a putative signal peptide (SP) and is expressed nearly 120 times more in infected leaves of wheat than in urediniospores^23^. Blastp showed that the candidate effector has 14 hypothetical homologues in *Pst*, *Puccinia graminis* f. sp. *tritici* and *Puccinia triticina*. We noted that PstSCR1 (also known as Pstha2a5^23^) protein sequence has three conserved (Y/F/W)x(C) motifs (Supplementary Fig. S1), one of which is located at the N-terminus as described in many wheat rust and other fungal effector candidates^29–32^. In order to test whether PstSCR1 is expressed during *Pst* infection of wheat, we employed qPCR (Supplementary Fig. S2) using infected samples collected at different time points (24-h, 72-h, 8-d and 10-d). The PstSCR1 was expressed highly between 72-h post-infection (hpi) to 8-d post-infection (dpi), but its expression was reduced at 10-dpi (Supplementary Fig. S3). Sequencing of the isolated PCR product at 8-dpi showed a perfect match with the reported EST sequence of PstSCR1. These results show that PstSCR1 family is exclusively conserved within three closely related *Puccinia* species.

### Apoplast targeted PstSCR1 enhances plant immunity against oomycete pathogens in *N*. *benthamiana*

**Figure 1:**
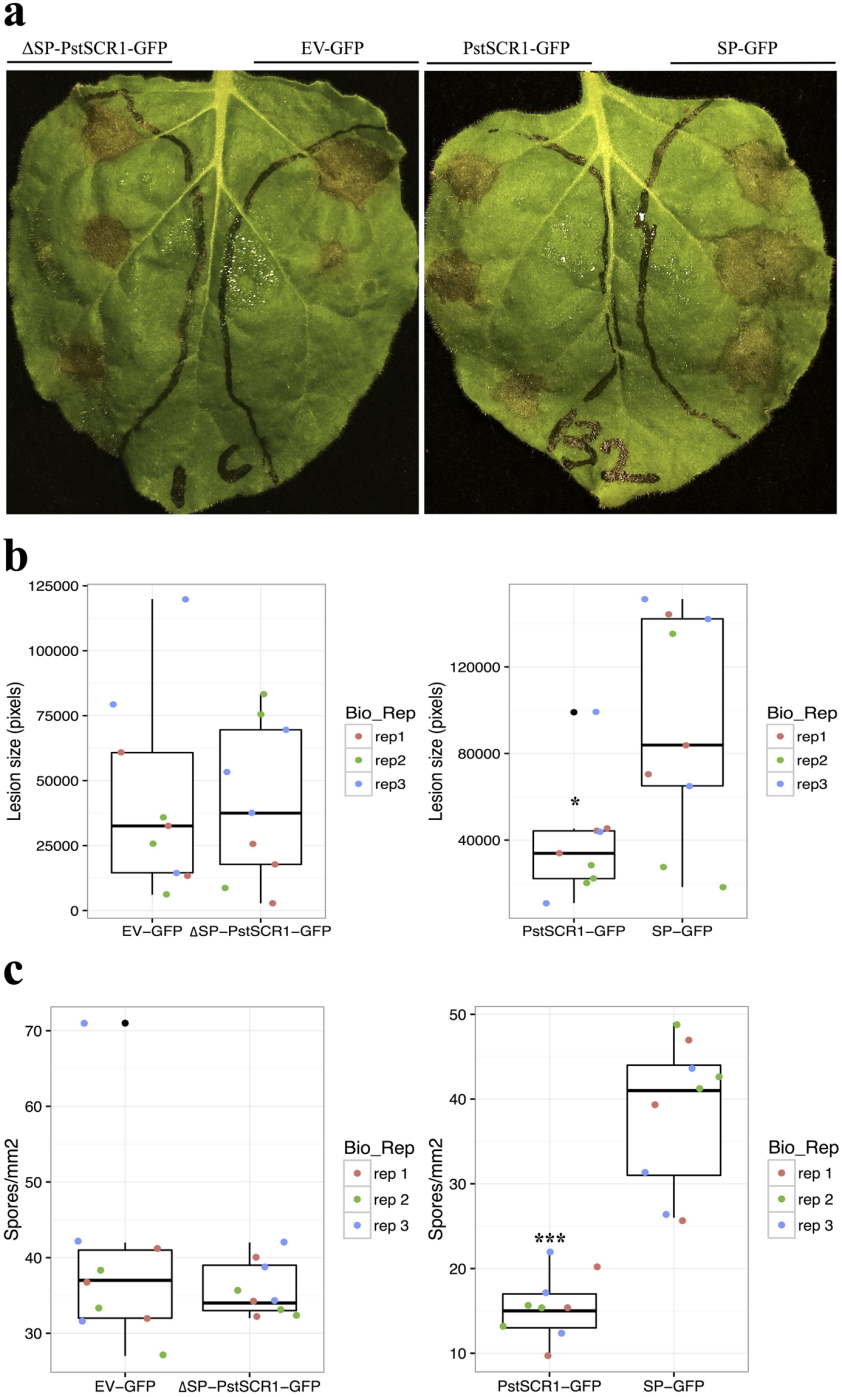
Expression of PstSCR1 reduces infection capacity of oomycete pathogens. (**a**) The infection of *N*. *benthamiana* leaf with *P*. *infestans* after expressing ΔSP-PstSCR1-GFP and PstSCR1-GFP constructs. Photographs were taken after 8-dpi. (**b**) *P*. *Infestans* lesion sizes were reduced in leaves expressing PstSCR1-GFP in a SP-dependent manner. Leaf patches expressing PstSCR1-GFP showed significantly smaller lesions compared to leaves expressing SP-GFP, whereas patches expressing ΔSP-PstSCR1-GFP showed similar lesion sizes to those expressing EV-GFP, as measured in pixels (by ImageJ tool). Asterisk indicates significant differences by ttest (*P ≤ 0.05). (**c**) *Peronospora hyoscyami f*. *sp*. *tabacina* spore count was reduced in leaves expressing PstSCR1-GFP in a SP-dependant manner. *N*. *benthamiana* leaves were infected with *P*. *tabacina* and spores were counted 8-dpi. Leaf patches expressing PstSCR1-GFP showed significantly less spores than patches expressing SP-GFP, whereas patches expressing ΔSP-PstSCR1-GFP showed similar spores to those expressing EV-GFP. Asterisks indicate significant differences by ttest (***P ≤ 0.001).

**Figure 2:**
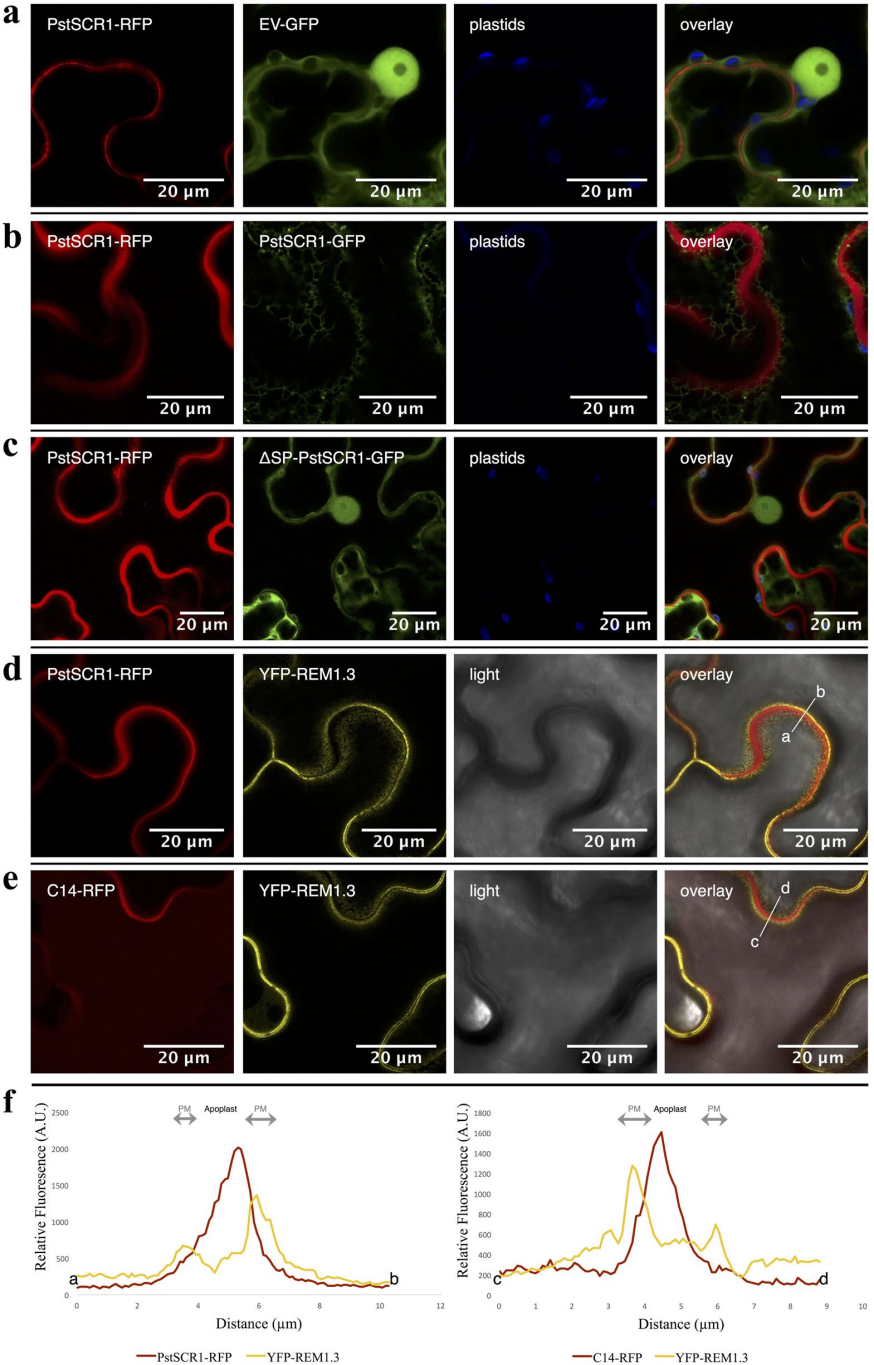
PstSCR1 accumulates in the plant apoplast. *N*. *benthamiana* plants were co-expressed by agro-infiltration using the following constructs: (**a**) pGWB454/PstSCR1-RFP and pK7FWG2/EV-GFP, (EV: empty vector), as nucleo-cytoplasmic marker; (**b**) pGWB454/PstSCR1-RFP and pK7FWG2/PstSCR1-GFP; (**c**) pGWB454/PstSCR1-RFP and pK7FWG2/ΔSP-PstSCR1-GFP. (**d**) pGWB454/PstSCR1-RFP and pK7/YFP-REM1.3. (**e**) pGWB554/C14 (Apoplast and vacuole marker^47^) and pK7WGY2/REM1.3 (Plasma membrane marker^﻿﻿58–﻿60^). (**d**) and (**e**) indicate PstSCR1 expressed with its SP accumulates in apoplastic space but not at the plasma membrane. (**f**) The intensity plots illustrate relative RFP and YFP fluorescence signals along the line connecting the points; a-b and c-d in overlayed images of (**d**) and (**e**), respectively.

To determine the extent to which PstSCR1 alters plant immunity, we expressed it in *N*. *benthamiana* leaves with its SP (PstSCR1) or without its SP (ΔSP-PstSCR1) and performed infection assays using the hemibiotroph *Phytophthora infestans* and an obligate biotroph *Peronospora hyoscyami* f. sp. *tabacina*. Expression of PstSCR1 targeted to the apoplast significantly reduced infection of both *P*. *infestans* (Fig. 1a and b) and *P*. *tabacina* (Fig. 1c) whereas expression of cytoplasmic ΔSP-PstSCR1 had no effect (Fig. 1). We validated the subcellular localizations of PstSCR1 and ΔSP-PstSCR1 using established subcellular markers in *N*. *benthamiana*. We showed that both RFP- and GFP-labeled PstSCR1 were secreted to the apoplast (Fig. 2), whereas ΔSP-PstSCR1 remained cytoplasmic (Fig. 2c).

Our immunoblot analysis of immunoprecipitates obtained from total protein extracts expressing PstSCR1 fusion constructs revealed expected sized fragments (Supplementary Fig. S4). Nevertheless, apoplastic PstSCR1 was more stable than cytoplasmic ΔSP-PstSCR1 possibly due to inefficient folding in the cytosol (Supplementary Fig. S4). These results suggest that PstSCR1 is an apoplastic effector whose expression in *N*. *benthamiana* results in enhanced disease resistance either due to its activation of surface immune receptors or adverse effects of its virulence function in a non-host plant.

### Overexpression of PstSCR1 induces cell death in *N*. *benthamiana*

**Figure 3:**
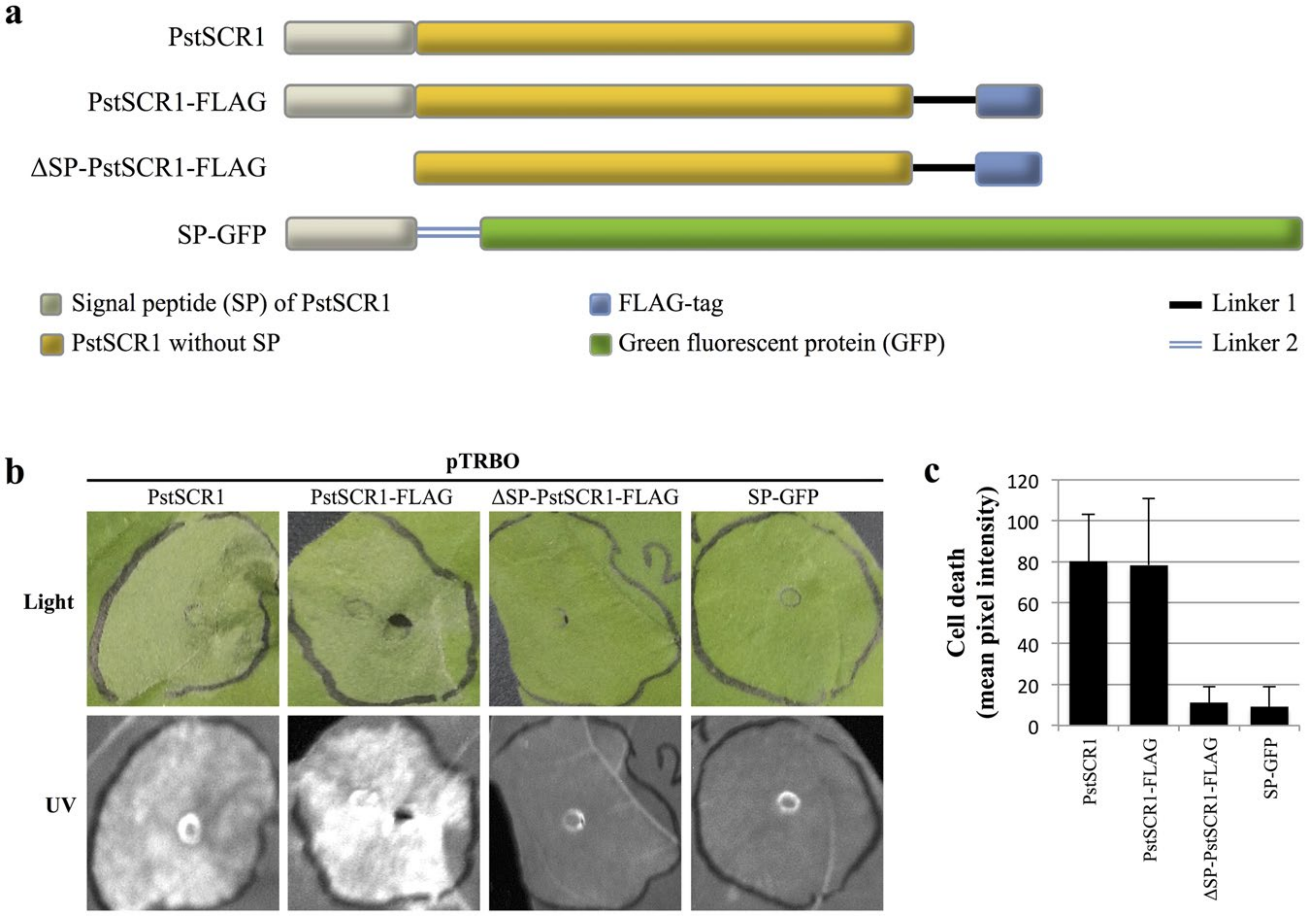
Secretion of PstSCR1 is required to induce cell death in *N*. *benthamiana*. (**a**) The schematic view of constructs used in the experiment. (**b**) PstSCR1 overexpressed the pTRBO vector with a secretion signal causes HR. Shown are representative *N*. *benthamiana* leaf patches expressing pTRBO/PstSCR1, pTRBO/PstSCR1-FLAG, pTRBO/ΔSP-PstSCR1-FLAG and pTRBO/SP-GFP, 4-dpai. (**c**) Cell death quantification of infiltrated leaf regions. Pixel intensities were normalized by subtracting background in non-infiltrated zone. Error bars represent standard deviations.

Next, to further determine the effect of PstSCR1 on plant immunity, we transiently overexpressed PstSCR1 in *N*. *benthamiana* with and without a FLAG-tag. Four days post agro-infiltration (dpai) we observed cell death, whereas overexpression of either ΔSP-PstSCR1 with a FLAG-tag, or GFP with a SP (SP-GFP), did not result in cell death (Fig. 3). Therefore the PstSCR1 secretion signal is not only indispensible for apoplastic targeting (Fig. 2) but also for its cell death–inducing activity (Fig. 3)^15, 33, 34^. We found that cell death was only observable with PstSCR1-FLAG when expressed in the strong pTRBO vector. Expressing PstSCR1 in pK7FWG2 vector did not generate any observable cell death (Supplementary Fig. S5).

**Figure 4:**
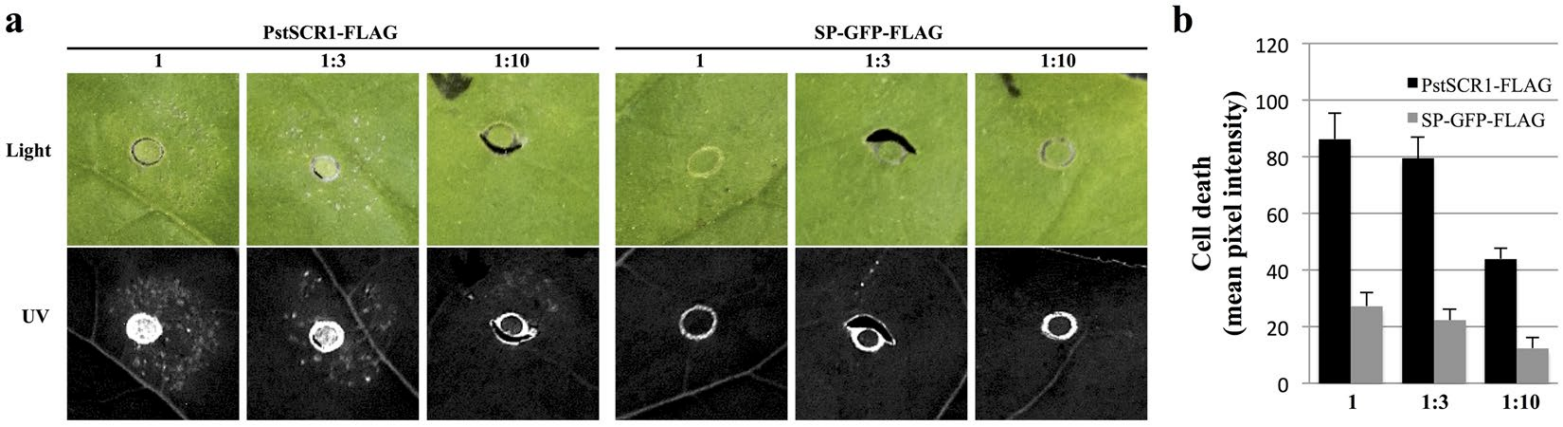
Apoplastic fluid containing the secreted PstSCR1 triggers cell death in *N*. *benthamiana* in a dose-dependent manner. The infiltration of *N*. *benthamiana* was conducted with apoplastic fluid containing processed PstSCR1. (**a**) Shown is a representative leaf of *N*. *benthamiana* infiltrated with apoplastic fluid (with no dilution (600–700 μg/μL), 1:3 and 1:10 dilutions in ddH_2_O) from *N*. *benthamiana* expressing PstSCR1-FLAG and SP-GFP-FLAG. Four days after apoplastic fluid infiltration, the leaves were examined under normal light and UV exposure. (**b**) Cell death quantification of infiltrated leaf regions. Pixel intensities were normalized by subtracting background in non-infiltrated zone. Error bars represent standard deviations.

To determine the extent to which apoplastic PstSCR1 stimulates cell death, we performed cell death assays using apoplastic extracts in the presence or absence of PstSCR1 (Fig. 4). We observed cell death in a dose dependent manner in leaves infiltrated with apoplastic fluid with PstSCR1, but not with the secreted GFP control (Fig. 4). We analysed apoplastic fluid and protein extract of remnant leaves that were used for apoplastic fluid isolation on immunoblots (Supplementary Fig. S6). The theoretical size of the PstSCR1-FLAG is smaller than the size observed on blots, which implies post-translational modification(s). In the control samples, Anti-GFP western blotting revealed that GFP is present both in apoplast and total protein extract. However, Anti-FLAG antibody did not detect SP-GFP-FLAG in the apoplastic extract, suggesting FLAG-tag was cleaved and SP-GFP-FLAG is prone to proteolysis in the apoplast (Supplementary Fig. S6). These results demonstrate PstSCR1 is stable in the apoplast, thus providing the possibility that it is functional.

### *NbBAK1* silencing leads to reduced cell death triggered by PstSCR1

**Figure 5:**
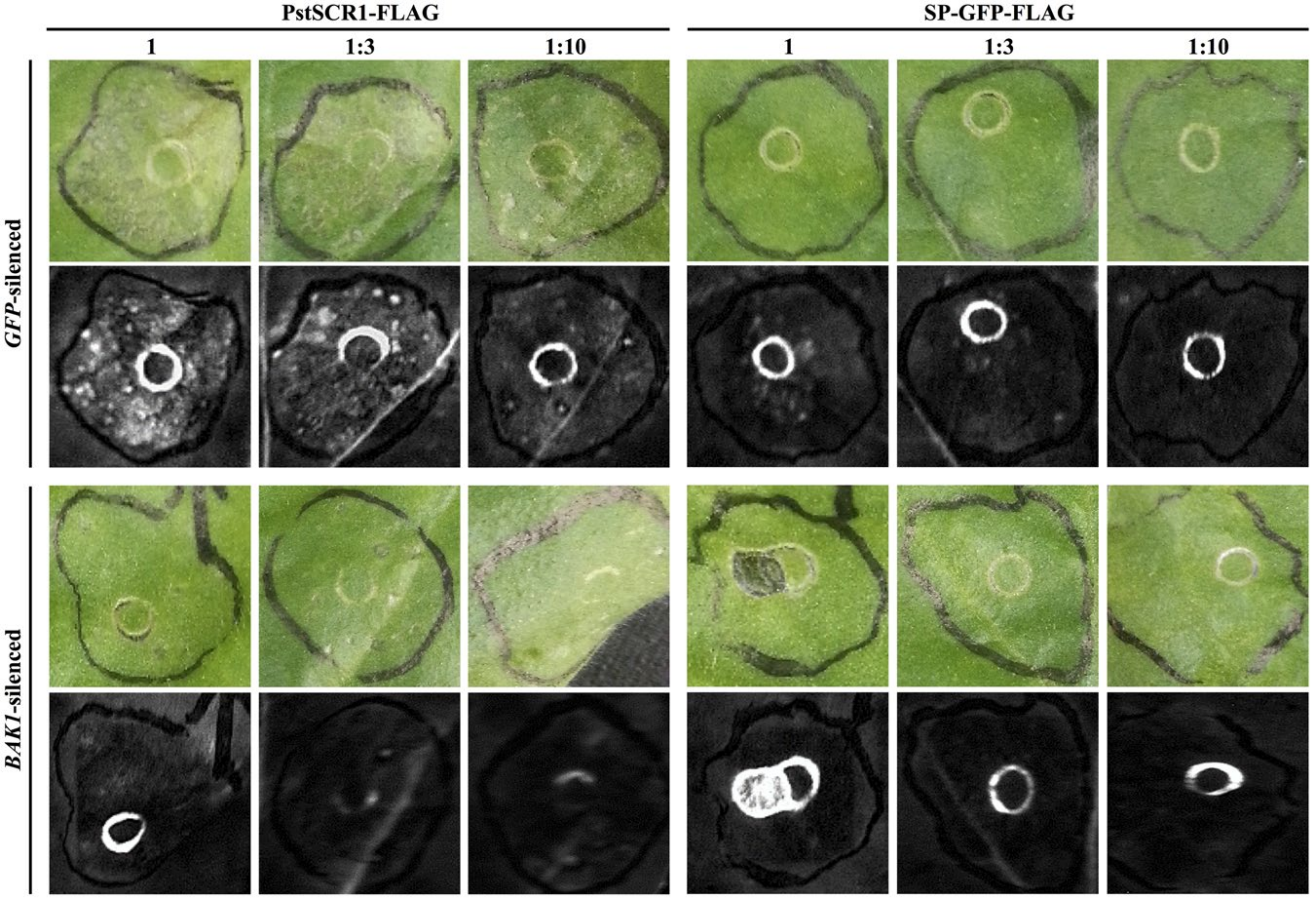
NbBAK1 is required for PstSCR1 triggered cell death. Apoplastic fluid from plant samples expressing PstSCR1-FLAG and SP-GFP-FLAG were infiltrated into *NbBAK1* and *GFP* silenced *N*. *benthamiana* with varying amounts; 1 ((no dilution), 1:3 and 1:10) dilutions. Following 4-d after apoplastic fluid infiltration, the leaves were examined under normal light and UV exposure.

The plant receptor-like kinase *SERK3*/*BAK1* is involved in response to PAMP molecules and is a key participant of the PTI response^35–37^. To check whether *SERK3*/*BAK1* is involved in cell death by PstSCR1, we tested *SERK3*/*BAK1*-silenced *N*. *benthamiana* plants with apoplastic fluid from tissue expressing secreted PstSCR1. In *SERK3*/*BAK1* silenced *N*. *benthamiana* leaves (Supplementary Fig. S7) cell death was decreased (Fig. 5). On the other hand, in the *GFP*-silenced samples no change was observed (Fig. 5). Therefore, this indicates that PsrSCR1 triggered cell death is a *BAK1-*dependent process, which presumably requires a surface immune receptor yet to be characterized.

### PTI marker genes are induced with PstSCR1 injection

To further illustrate that PstSCR1 triggers PTI-like responses, we decided to analyse its effect on defence-related gene up-regulation. We chose two *N*. *benthamiana* genes namely *NbCYP71D20* (a putative cytochrome P450) and *NbACRE31* (a putative calcium-binding protein), which are induced upon PAMP treatment^38, 39^. We purified PstSCR1 from the apoplastic fluid and infiltrated it into *N*. *benthamiana* leaves. The defence gene, *NbACRE31* was activated early, 2-d after purified PstSCR1 infiltration and remained stable^34^ at 4-d but the activation of *NbCYP71D20* took place later (4-d) (Supplementary Fig. S8). The expression of these defence genes was not detected in control leaves that were treated with SP-GFP-FLAG immunoprecipitated by Anti-FLAG from apoplastic fluid of *N*. *benthamiana* leaves.

## Discussion

The apoplast is a hostile environment and critical barrier for plant pathogens to overcome. Although remarkable progress has been made in identifying defence-related pathways targeted by pathogen effectors, the information on how pathogens pass the early barrier of plant immunity is very limited^40^. Here we report that a small, cysteine-rich effector-like protein PstSCR1 secreted by *Pst* triggers PTI responses at the cell surface. Consistent with this, heterologous expression of the PstSCR1 targeted to the extracellular space in *N*. *benthamiana* enhanced disease resistance against the oomycete pathogens *P*. *infestans* and *P*. *tabacina*. Thus, PstSCR1 secreted by the yellow rust fungus carries the characteristics of a proteinaceous PAMP that activates immunity in a non-host solanaceous plant *N*. *benthamiana*.

During infection of wheat with the *Pst-78* strain, *PstSCR1* gene expression was highly induced at 3-dpi, with peak expression observed at 8-dpi, which is consistent with previous gene expression studies of *PstSCR1* ^23^. The up-regulation of *PstSCR1* coincides with development of the fungal mycelium (2- to 8-dpi), in which generation of haustoria and haustorial mother cells, and formation of the pustule bed occurs for development of the uredinium and sporulation^41^. While we failed to detect *PstSCR1* gene expression at 24 hpi on the host plant wheat, we cannot exclude the possibility that it is expressed at early time points of infection on a non-host such as *N*. *benthamiana*. It is likely that multiple factors contribute to non-host resistance, and indeed other *Pst* effectors or secreted proteins may be involved. According to the phylogenetic tree we generated (Fig. S1B), the clade of PstSCR1 exclusively includes homologs from *Pst*. Moreover, from our database search, all PstSCR1 homologs found are conserved within closely related *Puccinia* species. Thus, PstSCR1 homologs may have evolved rapidly within *Puccinia* species.

Like apoplastic effectors, fluorescent fusion proteins of PstSCR1 show accumulation in the apoplast in a signal peptide dependent manner (Fig. 2)^15, 33, 42^. Expression of apoplast-targeted PstSCR1 in *N*. *benthamiana* increased resistance to oomycete pathogens, whilst cytoplasmic PstSCR1 did not (Fig. 1). The enhanced disease resistance against the oomycete pathogens triggered by PstSCR1 could be due to activation of PTI by PstSCR1 and/or direct competition between PstSCR1 and oomycete effectors for the host susceptibility factors. In line with this, purified PstSCR1 triggered defence related gene expression, which is one of the hallmarks of the PTI responses. Thus, our results are consistent with the view that PstSCR1 is a secreted rust protein that triggers PTI responses.

When highly expressed by the tobacco mosaic virus (TMV) based pTRBO vector^43^, full length PstSCR1 triggered cell death but not in the absence of its signal peptide. The cell death did not occur when PstSCR1 was expressed by the weaker 35S promoter (Fig. S5), however it was sufficient to limit oomycete infections (Fig. 1). The cell death triggered by higher expression of PstSCR1 can be explained by two possibilities: Firstly, over production of PstSCR1 could hyper activate the PTI machinery resulting in HR like cell death. Secondly, PstSCR1 might show a toxic effect due to its overrepresentation in the apoplast perhaps by non-specifically perturbing the extracellular environment. However, our results favour the former, as silencing of *NbBAK1*, one of the main components of the PTI signalling pathways, compromised cell death induced by PstSCR1 (Fig. 5). Thus, the cell death phenotype observed with higher gene expression of PstSCR1 is most likely due to over activation of the PTI signalling pathway.

Our results suggest that PstSCR1 is recognized in non-host plant *N*. *benthamiana* by an undetermined surface immune receptor that requires NbBAK1 for signalling. The finding that the oomycetes were incapable of fully suppressing this immune response indicates that this could be a divergent PTI pathway that cannot be suppressed by the oomycete effectors effectively. This would explain the increased resistance against oomycete pathogens stimulated by PstSCR1 when expressed at low levels that does not activate HR.

The molecular mechanisms that mediate non-host resistance are poorly characterized. It is likely that multiple factors affect this phenomenon. Our results demonstrate that pathogen secreted proteins such as PstSCR1 might fail at immune evasion in non-host plants and contribute to non-host resistance rather than serving as virulence cues. In line with this, Kettles *et al*., showed that effector candidates from the wheat pathogen *Zymoseptoria tritici* are recognized in *N*. *benthamiana* ^34^. Previous studies showed that many PRRs strongly associate with NbBAK1 upon ligand binding. Recently, Saur *et al*., demonstrated that BAK1 can be used as a bait to identify PRRs upon ligand activation^44^. Thus, elicitors like PstSCR1 can be exploited as tools to discover novel immune receptors that mediate non-host resistance. Interfamily transfer of plant PRRs were proven to be effective to engineer enhanced disease resistance^45^. Therefore, once identified, the PRR that responds to PstSCR1 in *Nicotiana* can be transferred to wheat plants to improve disease resistance.

## Materials and Methods

### Bioinformatics tools

The cDNA sequence of the candidate effector was obtained from National Center of Biotechnology Information (NCBI) with EST accession number of GH737102. The open reading frame (ORF) of the EST was predicted by ORF-Finder of NCBI (http://www.ncbi.nlm.nih.gov/gorf/gorf.html). The signal peptide (SP) sequence was determined by SignalP 4.1 online tool (http://www.cbs.dtu.dk/services/SignalP/)^46^. The amino acid sequence was analysed *via* blastp at the NCBI to detect the closely related sequences.

### Cloning

The primers used in the study are listed in Table S1, presented in supplementary information. The vectors and the constructs, pTRV1, pTRBO, pTRBO/GFP and pTRBO/FLAG-RFP, were gifts from Kamoun Lab, Sainsbury laboratory, Norwich, UK. The vectors (pGWB554/C14 and pK7WFY2/REM1.3) expressing C14:RFP and YFP:REM1.3 were as in Bozkurt *et al*. and Raffaele *et al*., respectively^47, 48^. The PstSCR1 gene had made synthesized with SP, and *Pac*I and *Not*I restriction site extensions corresponding to N-terminus and C-terminus, respectively and the construct was obtained as pBSK/PstSCR1 (GeneScript). For the subcellular localization of PstSCR1 experiments, the gene was re-amplified with and without SP from pBSK/PstSCR1 with CACC-SP or CACC-ATG-SCR1 (without SP) as forward primer and SCR1-noSTP as reverse primer and cloned into pENTR/D-TOPO vector (Invitrogen) and recombined with the two different destination vectors; pK7FWG2^49^ and pGWB454^50^ through LR clonase reaction (Invitrogen). For the overexpression, the effector with (PstSCR1) and without SP (ΔSP-PstSCR1) and FLAG-Tag on the C-terminus was constructed by PCR using forward primers; PacI-SP-fw or PacI-noSP-SCR1-fw and reverse primers; SCR1C-FLAGRev2 and SCR1C-FLAGRev1. The final PCR product was obtained with *Pac*I restriction site on the 5′-end and FLAG-Tag and *Not*I restriction site on the 3′-end. The PCR products were cloned into pTRBO (pJL48) vector^43^ and labelled as pTRBO/Pst SCR1-FLAG and pTRBO/ΔSP-PstSCR1-FLAG. To express secreted GFP (SP-GFP), SP was amplified with CACC-SP and SP-noSTP primers using pBSK-PstSCR1 as a template; then, cloned into pENTR/D-TOPO, and followed by LR recombination (Invitrogen) into pK7FWG2^49^ and labelled as pK7FWG2/SP-GFP. For the cloning of SP-GFP-FLAG, SP-GFP was first amplified with PacI-SP-fw and GFP-FLAG-Rev primers using pK7FWG2/SP-GFP as a template. SP-GFP-FLAG was generated using the amplified product as a template with the primers PacI-SP-fw and SCR1-C-FLAGRev1 as forward and reverse primers, respectively. The SP-GFP-FLAG was cloned into pTRBO (pJL48)^43^ and denoted as pTRBO/SP-GFP-FLAG. For the cloning and the amplification of the plasmids, the constructs were maintained in *E*. *coli* Top10 strain. In Agro-infiltration experiments, the constructs were introduced into *Agrobacterium tumefaciens* GV3101 strain by electroporation.

### Agro-infiltration assays

*Nicotiana benthamiana* plants were grown at 20–24 °C with 16-light/8-dark cycle in a growth room. Four to six week-old plant middle leaves were used for agro-infiltration assays. Agrobacterium-mediated gene transfer was conducted with minor modifications as described elsewhere^51^. *A*. *tumefaciens* (GV3101, pMP90) culture was pelleted by 5 min, at 4000 rpm in room temperature (RT) and the cell pellet was washed with distilled water, washing was repeated two more times. The cells were suspended in Agro-induction medium (10 mM MES pH 5.6, 10 mM MgCl_2_). The concentration of the suspension was adjusted to 0.2 A_600_ for infiltration. The infiltrated leaves were collected after 2–4 days post-agroinfiltration (dpai) depending on the expression level for microscopic imaging, apoplastic fluid isolation and total protein extraction.

### Apoplastic fluid isolation

The *Agrobacterium* (with pTRBO/PstSCR1-FLAG or pTRBO/SP-GFP-FLAG) infiltrated *N*. *benthamiana* leaves of 2–3 dpai were used to obtain apoplastic fluid as in the method previously described^52^. Briefly, the leaves were detached, rinsed with distilled water, carefully folded, placed in a 60-mL syringe filled with distilled water, and vacuum was applied for 5–15 seconds repeatedly until the leaves appeared as dark translucent. The leaves were wiped with clean tissue or filter paper, and sandwiched in parafilm sheets, rolled and placed in a 20-mL syringe, centrifuged in 50 mL falcon tube for 10 min at 1,000 g, at 4 °C. The collected apoplastic fluid (400–500 μL/1.0–1.5 g leaf sample) was centrifuged at 15,000 g for 5 min. The supernatant, having 600–700 μg/μL total protein concentration, was transferred to a fresh tube on ice and stored at −80 °C later use or utilized on the same day.

### Apoplastic fluid infiltration

Apoplastic fluid samples obtained from various constructs containing Agrobacterium-infiltrated *N*. *benthamiana* were infiltrated into fresh *N*. *benthamiana* leaves until the injected area reaches to the size of a penny. The samples and the controls were infiltrated side by side on the same leaf with no or various dilutions (1 (no dilution), 1:3 and 1:10) in ddH_2_O. The presence of or the level of hypersensitive response (HR) was examined 4–5 days after apoplastic fluid infiltration.

### PstSCR1 purification

Anti-FLAG M2 affinity gel (Sigma, A2220) was mixed gently by pipetting with a cut tip. After resin completely suspended, 50 (or 250 for large scale) μL of it was placed into a 50 mL falcon tube containing 250 μL (25 mL for large scale) apoplastic fluid or protein extract diluted (1:1) with IP buffer ((10% glycerol, 25 mM Tris, pH 7.5, 150 mM NaCl, 1 mM EDTA) freshly mixed, 0.1% (v/v) Tween 20). The tube was mixed turning end-over-end at 4 °C for 3-hour. After the incubation, resin was pelleted at 800 g for 30 sec, and supernatant was removed; then pellet was re-suspended in 1 mL IP buffer and transferred into fresh microcentrifuge tube. The wash was repeated four times. After last wash, the remnant liquid was removed very carefully by using syringe with needle not to remove the beads. Immobilized proteins were eluted from the beads in re-suspension solution of 500 μL IP buffer containing 150 ng/μL FLAG tag peptide and the tube was gently shaken in horizontal position for 2-hour, at 4 °C. Eluted proteins (supernatants) were transferred into fresh tube.

### Infiltration of purified PstSCR1 into *N*. *benthamiana*

Apoplastic-purified PstSCR1 samples were diluted (1:10) with ddH_2_O and injected into fresh *N*. *benthamiana* leaves until the whole leaf infiltrated. As a control, immunoprecipitation sample obtained from apoplastic fluid of *N*. *benthamiana* expressing SP-GFP-FLAG was used. Two and four days after purified PstSCR1 protein infiltration, the expression levels of *NbCYP71D20* and *NbACRE31* were determined by qPCR.

### Virus induced gene silencing of *NbBAK1*

*NbBAK1* was silenced using the agrobacterium-mediated co-infiltration of the clones containing *Tobacco Rattle Virus* as pTRV2/BAK1 and pTRV1 with A_600_ ratio of 2:1 (0.4:0.2, respectively). As a viral control, Agro-pTRV2/GFP:pTRV1 (2:1) were co-infiltrated^53^. The newly emerged leaves of three weeks post-silenced tobacco were used for the injection of apoplastic fluid obtained from PstSCR1 expressing leaves.

### Infection assay

The effect of PstSCR1 expression during pathogen growth was assayed by transiently expressing pK7FWG2/PstSCR1-GFP on one half and pK7FWG2/SP-GFP on the other half of *N*. *benthamiana* leaves (4–5 weeks old). Following 4–5 hour post-infiltration (hpi), leaves were detached and either *Phytophthora infestans* 88069 or *Peronospora hyoscyami f*. *sp*. *tabacina* were inoculated as 3 spots of 10 μL of cultures of each, into each half of the infiltrated area of the leaves^54, 55^. The lesion diameter of *Phytophthora infestans* growth and the number of spores of *Peronospora hyoscyami f*. *sp*. *tabacina* were recorded at 8-dpi. The infection assay experiments were repeated on at least three independent *N*. *benthamiana* leaves.

### Immunoblotting

*N*. *benthamiana* leaves were grinded in liquid nitrogen, and 1 g of leaf powder was dissolved in a 2-mL extraction buffer GTEN (10% glycerol, 25 mM Tris, pH 7.5, 150 mM NaCl, 1 mM EDTA) freshly mixed with 2% (w/v), polyvinylpolypyrrolidone, 1X protease inhibitor, (Thermo #88666) and 10 mM dithiothreitol, 0.1% (v/v) Tween 20. The cell debris was pelleted by centrifugation and supernatant was collected in a fresh tube (first with 3,000 g for 10 min, and again twice with 21,000 g for 10 min in microcentrifuge at 4 °C). For western blot analysis, anti-FLAG (Thermo MA1-91878) and anti-GFP (Thermo MA5-15256) antibodies were used as the primary, and anti-mouse Alkaline Phosphatase conjugated antibody (Chemicon International #AP308A) was used as the secondary antibody.

### Confocal microscopy

*N*. *benthamiana* leaves after 2–3 dpai were cut in small pieces, immersed in distilled water and imaged on Leica 385 TCS SP5 confocal microscope (Leica Microsystems, Germany). The excitation of GFP, YFP and RFP probes were performed by 488, 514 and 561 nm laser diodes, respectively, and fluorescent emissions were detected at 495–550 (GFP and YFP) and 570–620 nm (RFP). For chloroplast autofluorescence, far infrared (>800 nm) excitation and emission were used.

### qPCR

The total RNA of 100 mg leaf sample was extracted using RNeasy Mini Kit (Qiagen). The first strand cDNA was synthesized in 20 μL reaction volume from 800 ng total RNA using Transcriptor First strand cDNA synthesis Kit (Roche) according to the instructions suggested by the manufacturer. qPCR was conducted using AccuPower GreenStar^TM^ qPCR Premix (BIONEER) with 10 μL of 1:20 diluted cDNA. To quantitative analysis of *NbSERK3*/*BAK1*, *NbCYP71D20* and *NbACRE31*, combinations of NbSerk3-qRT-F and NbSerk3-qRT-R, NbCYP71D20-F and NbCYP71D20-R, and NbACRE31-F and NbACRE31-R primers were used, respectively^56^. EF1α-qRT-F and EF1α-qRT-R were used amplification of Elongation factor 1 alpha gene (*EF1α*), used as constitutively expressed reference gene^15, 57^. PstSCR1-SP-5-UTR-F and PstSCR1-Rev primers used to amplify the PstSCR1 transcripts. The primer sequences used in qPCR amplifications are illustrated in Supplementary Table S1.

## Electronic supplementary material

Supplementary information
