# Supplementary material for: A Puccinia striiformis f. sp. tritici secreted protein activates plant immunity at the cell surface
Source: Sci Rep. 2017 Apr 25;7:1141. doi: 10.1038/s41598-017-01100-z (PMC5430700; doi:10.1038/s41598-017-01100-z)
Supplement: Supplementary file 1 — Supplementary information [file 41598_2017_1100_MOESM1_ESM.pdf]

A *Puccinia striiformis* f. sp. *tritici* secreted protein activates plant immunity at the cell surface

Bayantes Dagvadorj, Ahmet Caglar Ozketen, Ayse Andac, Cian Duggan, Tolga Osman Bozkurt, and Mahinur S. Akkaya

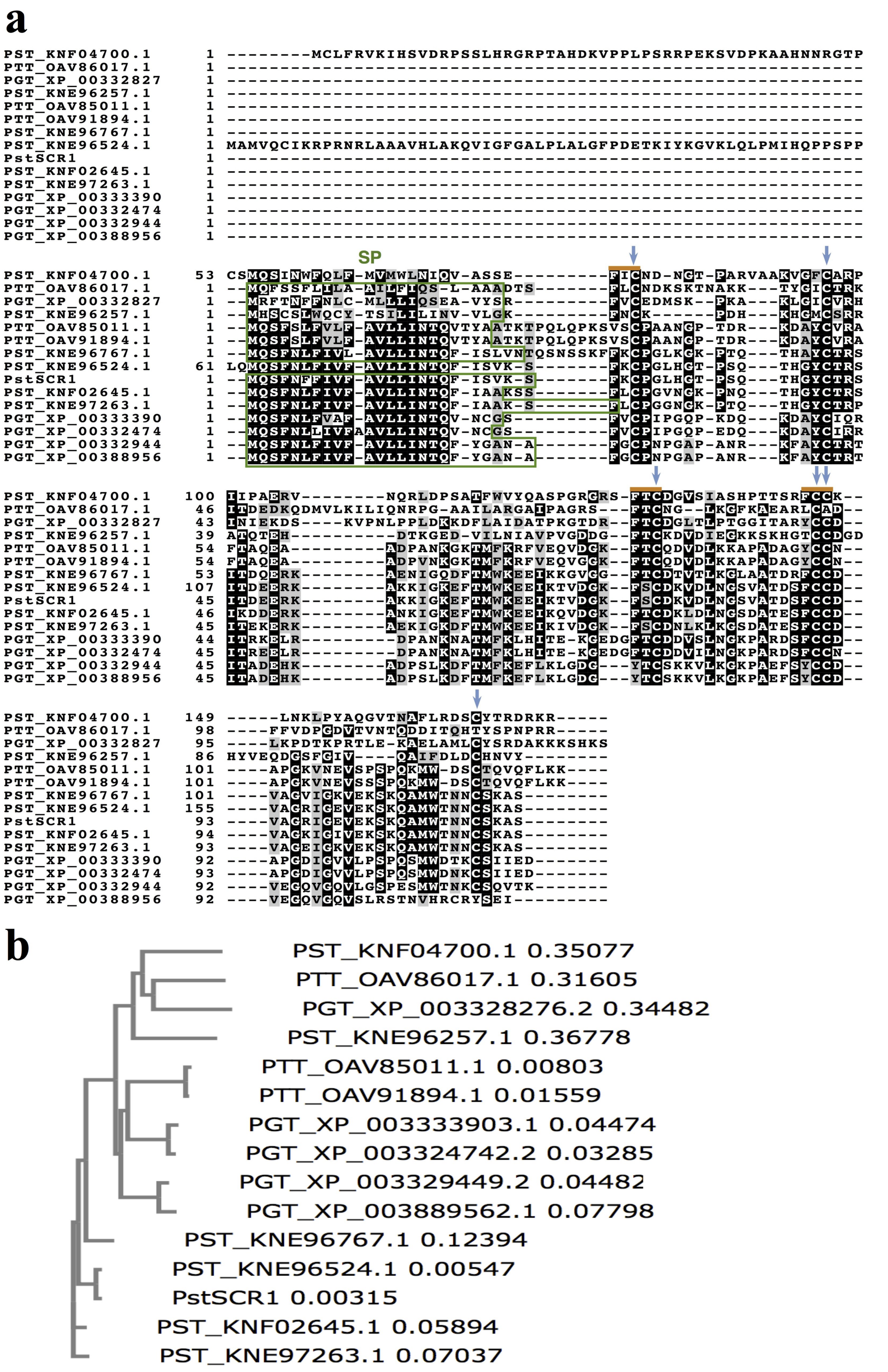

Figure S1. The Multiple sequence alignment and the phylogeny tree of PstSCR1 homolog proteins. a) Alignment was obtained using MUSCLE and displayed by BoxShade 3.21. Black letters represent identical amino acids. The predicted signal peptide (SP) sequences of PstSCR1 and its homolog proteins in green boxes. SP was not predicted in PST_KNF04700.1 and PST_KNE96524.1 due to extended N-termini. (Y/F/W)xC motifs and C residues are indicated with orange and blue arrows, respectively. **b)** The Phylogeny tree was constructed with Neighbour Joining without distance corrections.

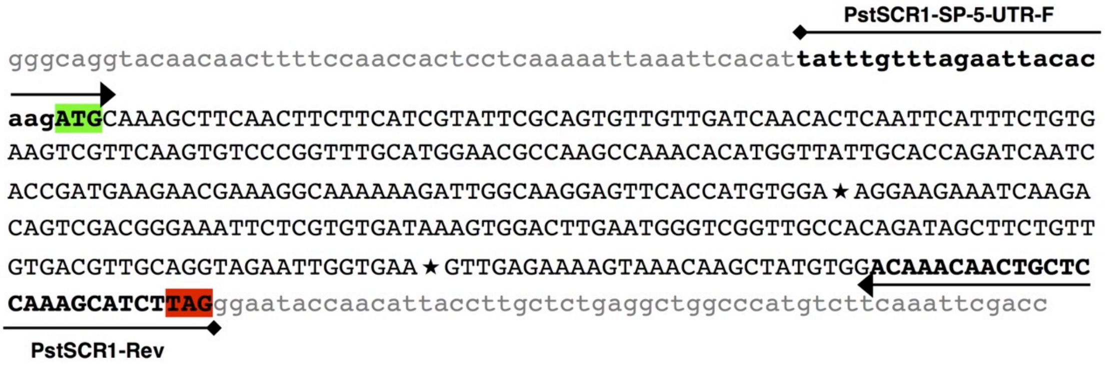

Figure S2. PCR primer design strategy to detect the presence of PstSCR1 expression in *Pst* infected wheat samples. PstSCR1 EST sequence; arrows indicate primers annealing regions and expected amplification size is 375 bp. Stars indicate the exon joints. The forward primer of PstSCR1 was designed to amplify the 5’ UTR region of the PstSCR1, which is predicted as the intron region in the closest homolog of PstSCR1.

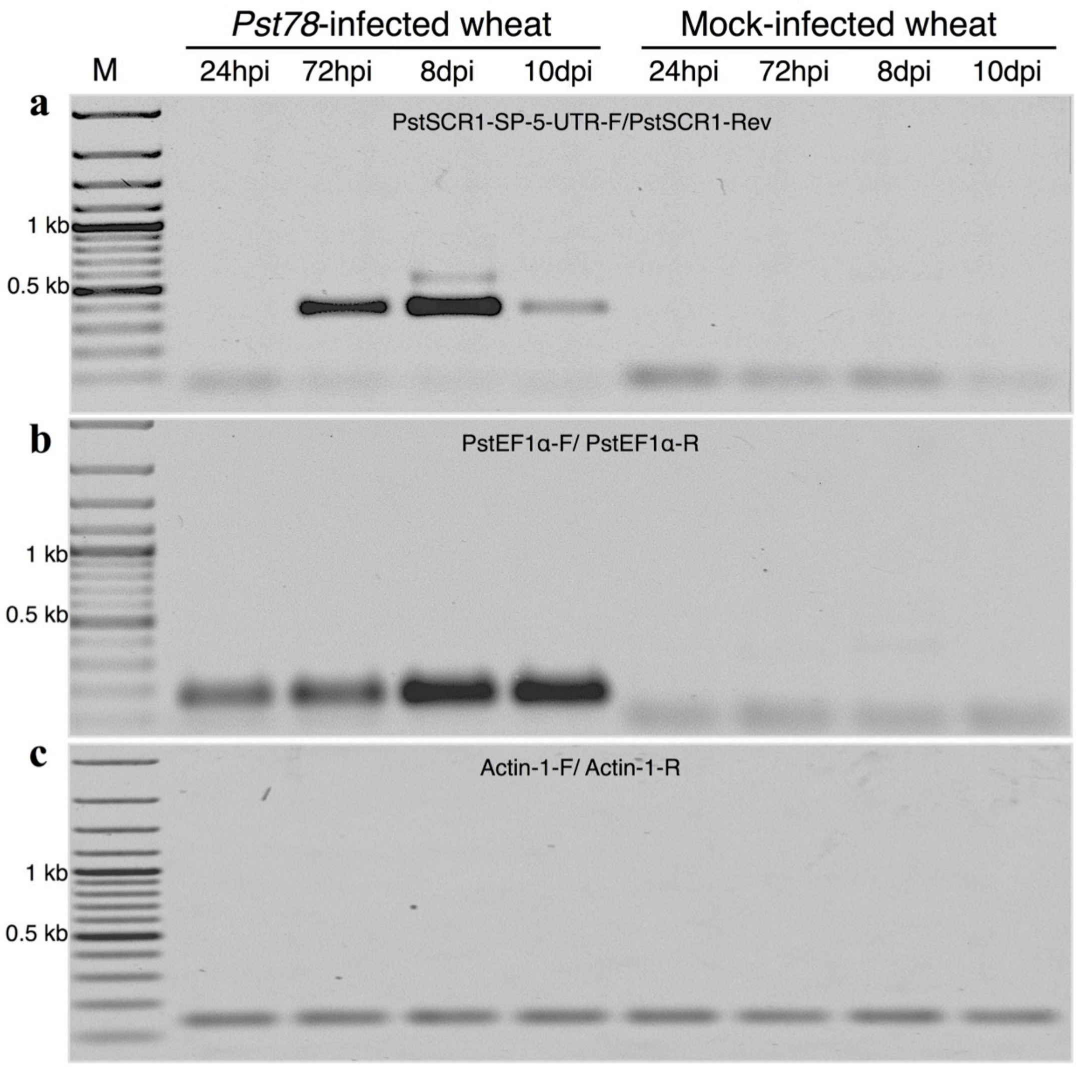

Figure S3. The PstSCR1 gene expression profile during *Pst-78* infection in wheat. **a**, **b** and **c** are PstSCR1 (exp: 375 bp), *Pst* Elongation factor 1 alpha (*PstEF1α*)[1], and wheat *Actin-1*[2] genes amplifications, respectively. qPCR products of 10 μL were loaded on 1% agarose gel. **M:** 100 bp ladder (Fermentas SM#0321).

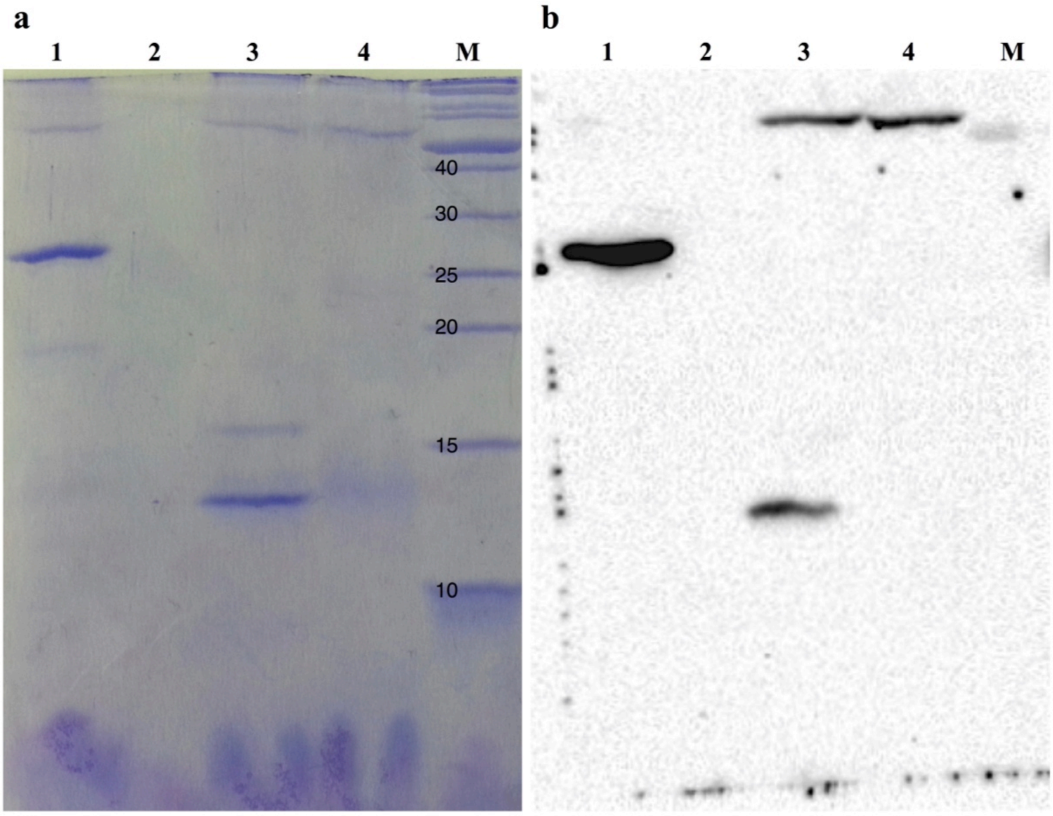

Figure S4. The PstSCR1 was purified by immunoprecipitation *in planta*. a) Anti-FLAG immunoprecipitation samples separated with SDS-PAGE (12 %) and stained with Coomassie Blue **b)** Immunoblot with Anti-FLAG antibody **1)** pTRBO/FLAG-RFP, **2)** No load **3)** pTRBO/PstSCR1-FLAG, **4)** pTRBO/ΔSP-PstSCR1-FLAG, and **M)** Protein marker (Pierce #PI-26614).

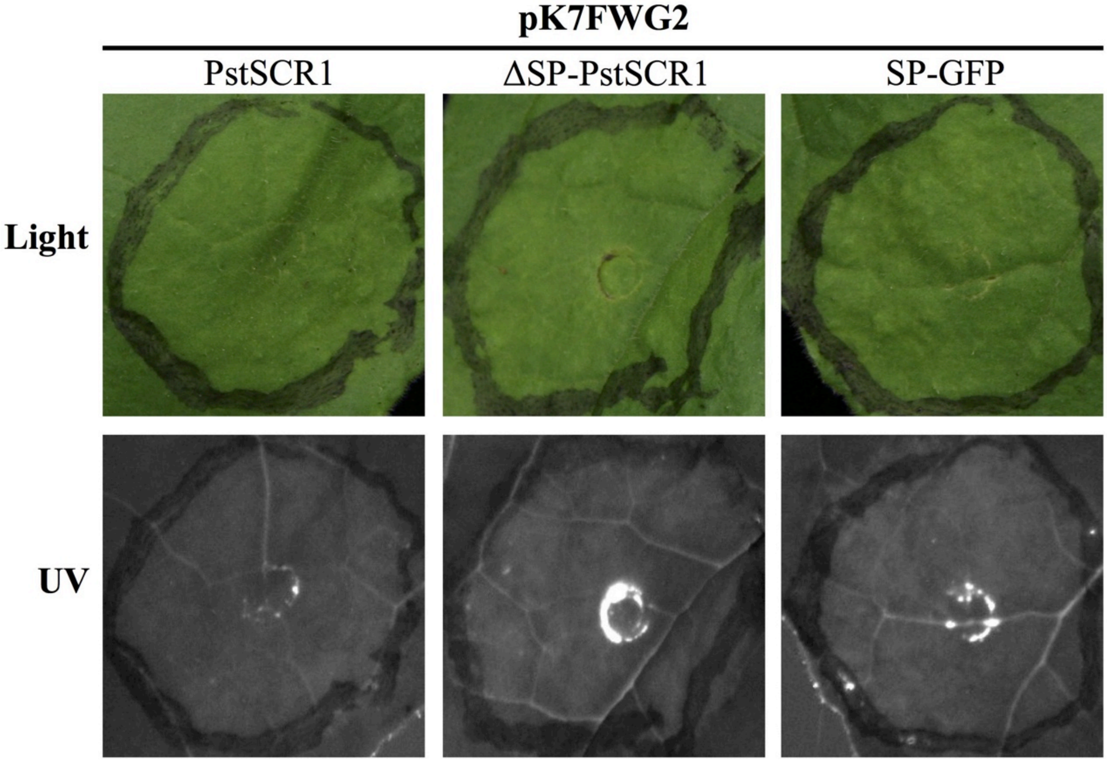

Figure S5. **PstSCR1 does not induce cell death in *N. benthamiana* when expressed with pK7FWG2.** A representative of six biological replicates: agroinfiltrated spots of *N. benthamiana* leaf expressing pK7FWG2/PstSCR1, pK7FWG2/ΔSP-PstSCR1 and pK7FWG2/SP-GFP, 4-dpai.

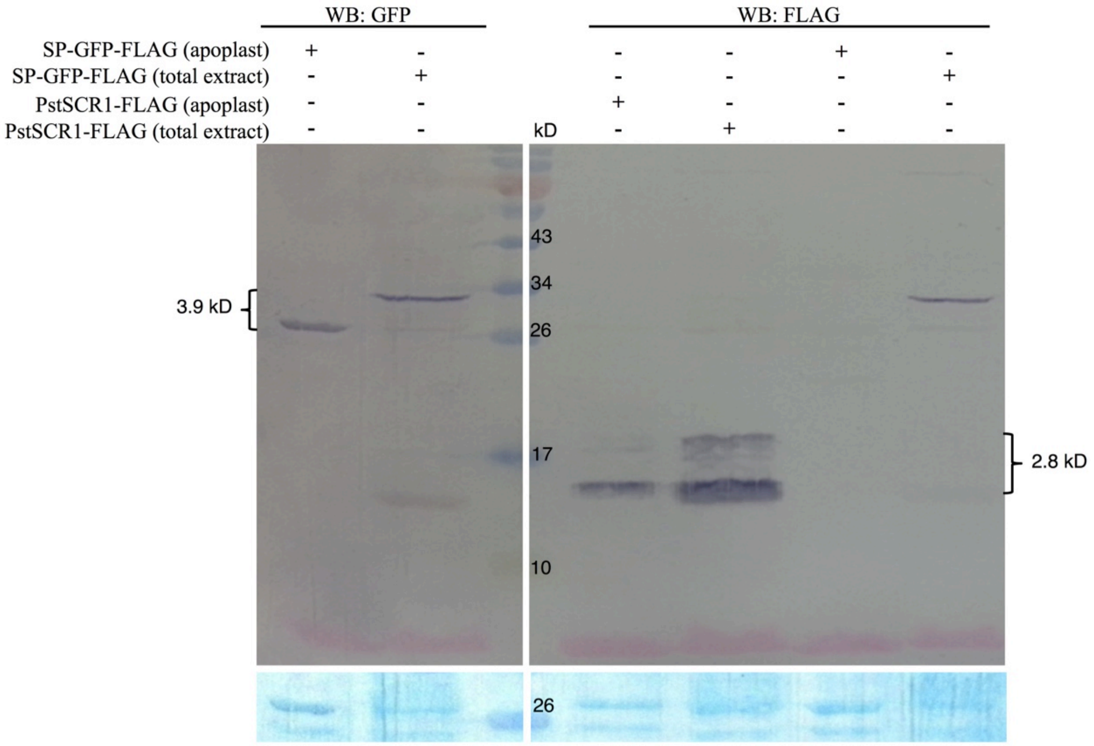

Figure S6. Immunoblot analysis of PstSCR1 fused with FLAG tag transiently expressed in *N. benthamiana*. Apoplastic fluid and total protein extract, from leaves after removal of apoplastic fluid, (10 µL) were loaded on SDS-PAGE gel and Western blotting was carried out with Anti-FLAG (Thermo) or Anti-GFP (Thermo) antibodies; total protein was detected by Reversible Protein Stain on PVDF membrane (Thermo). As a control, SP-GFP-FLAG was used. The theoretical sizes of the bands; SP-GFP-FLAG is 33.2 kD; SP-GFP-FLAG (SP and FLAG cleaved) is 29.4kD; PstSCR1-FLAG is 14.74 kD and ΔSP-PstSCR1-FLAG is 11.96kD.

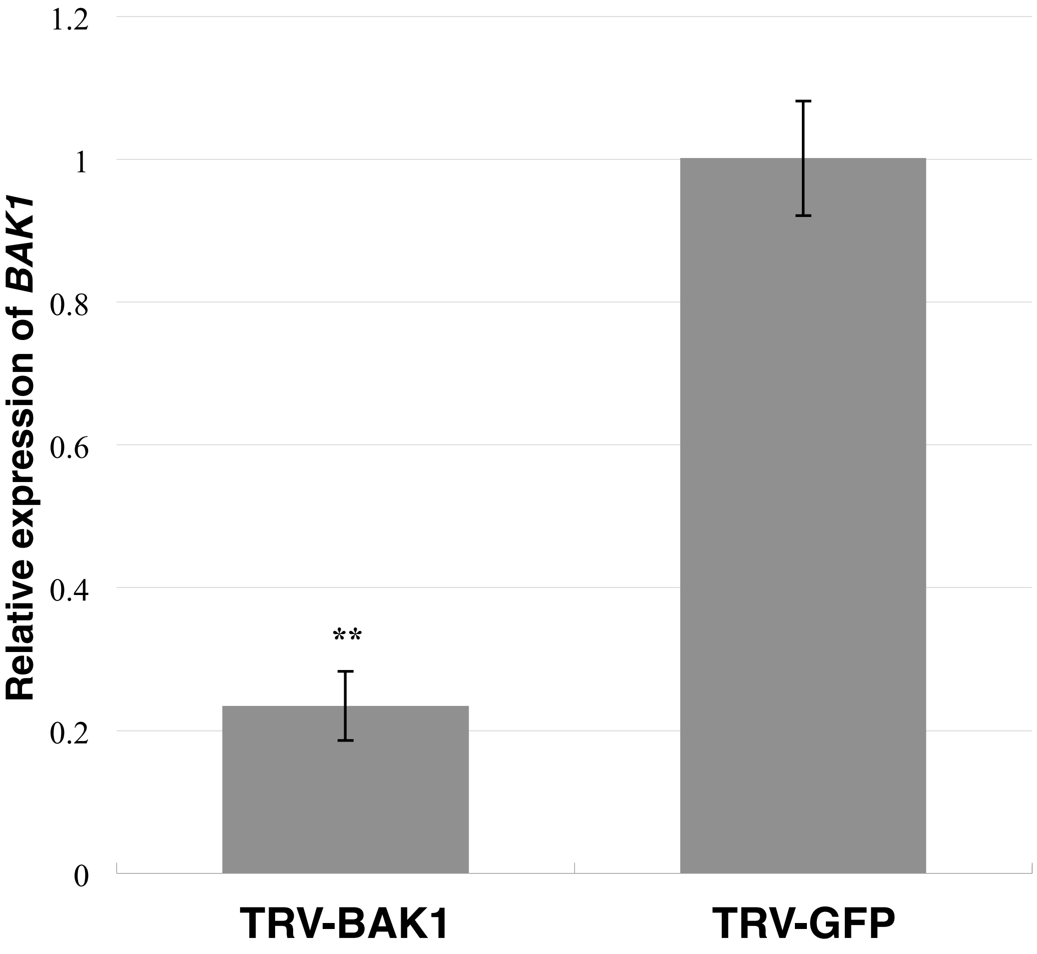

Figure S7. *NbBAK1* expression level determined after VIGS by qPCR. In *N. benthamiana* plant leaves, *NbBAK1* was silenced using TRV-BAK1 construct and TRV-GFP construct was used as a control for silencing[3]. The expression levels of *NbBAK1* were determined by qPCR in both plant sets. *EF1α* was used as an endogenous gene control. Relative expression levels were analysed by $2^{-\Delta\Delta Ct}$ method[4]. Means and standard deviations from biological replicates are illustrated. Asterisks indicate significant differences by ttest (**P ≤ 0.01).

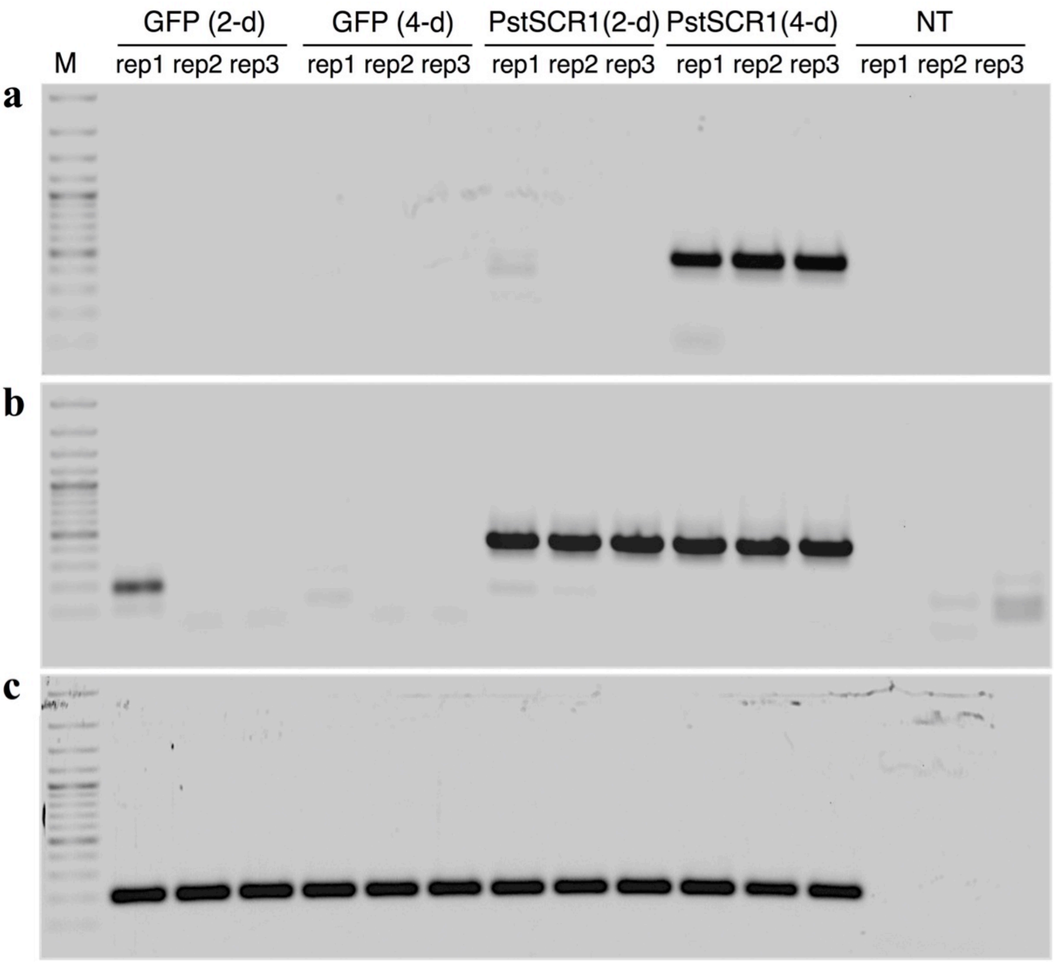

Figure S8. The PstSCR1 treated plants analysed by qPCR show defence related gene activation. Total RNA was isolated 2- and 4-d after purified PstSCR1 protein infiltration. The synthesized cDNAs were subjected to PCR using **a)** *NbCYP71D20,* **b**) *NbACRE31* or **c**) housekeeping *EF1α* gene specific primers and visualized in an agarose gel. **M:** 100 bp ladder (Fermentas SM#0321) and **NT**: no template PCRs.

Primer names	Sequence 5' to 3'	Length (bp)
PstSCR1-SP-5-UTR-F	tatttgtttagaattacacaagatg	25
PstSCR1-Rev	ctaagatgctttggagcagttgtttgt	27
PstEF1α-F	ttcgccgtccgtgatatgagacaa	24
PstEF1α-R	atgcgtatcatggtggtggagtga	24
Actin-1-F	aatggtcaaggctggtttcgc	21
Actin-1-R	ctgcgcctcatcaccaacata	21
CACC-SP	caccatgcaaagcttcaacttcttcatcg	29
CACC-ATG-SCR1	caccatgttcaagtgtcccggtttgcat	28
SCR1-noSTP	agatgctttggagcagttgtttgt	24
SP-noSTP	cgacttcacagaaatgaattgag	23
PacI-SP-fw	gggttaattaaatgcaaagcttcaacttcttcatcgtattcgcag	45
PacI-noSP-SCR1-fw	gagttaattaaatgttcaagtgtcccggtttgcatgg	37
SCR1C-FLAGRev2	ccttgtagtcggaattctcgagaagcttgacagatgctttggagcagttgt	51
SCR1C-FLAGRev1	attgcggccgcctatttgtcatcgtcgtccttgtagtcggaattctcgaga	51
GFP-FLAG-Rev	ccttgtagtcggaattctcgagaagcttgaccttgtacagctcgtccatgcc	52
NbSerk3-qRT-F	gcttcctgaggctgaataata	21
NbSerk3-qRT-R	gaagaagaagatgagggtgtag	22
NbEF1α-qRT-F	ctacctcaagaaggttggatac	22
NbEF1α-qRT-R	aacatcctgaagtggaagac	20
NbCYP71D20-F	accgcaccatgtccttagag	20
NbCYP71D20-R	cttgccccttgagtacttgc	20
NbACRE31-F	cgtcttcgtcggatcttcg	19
NbACRE31-R	ggccatcgtgatcttggtc	19

Table S1. Primers used for cloning and qPCR.

References

1. Yin, C. *et al.* Generation and analysis of expression sequence tags from haustoria of the wheat stripe rust fungus Puccinia striiformis f. sp. Tritici. *BMC Genomics* **10,** 626 (2009).
2. Bozkurt, O. Determination of Genes Involved in the Yellow Rust. (Middle East Technical University, 2007). http://etd.lib.metu.edu.tr/upload/12608246/index.pdf
3. Chaparro-Garcia, A. *et al.* The receptor-like kinase serk3/bak1 is required for basal resistance against the late blight pathogen Phytophthora infestans in Nicotiana benthamiana. *PLoS One* **6,** e16608 (2011).
4. Livak, K. J. & Schmittgen, T. D. Analysis of relative gene expression data using real-time quantitative PCR and the 2^-ΔΔCT Method. *Methods* **25,** 402–408 (2001).
